# Aberrant Salience, Psychotic-Like Experiences, and Anxiety: a Case-Control Study

**DOI:** 10.1192/j.eurpsy.2024.295

**Published:** 2024-08-27

**Authors:** G. P. Merola, A. Patti, D. Benedetti, B. Bozza, A. Ballerini, V. Ricca

**Affiliations:** ^1^Psychiatry; ^2^University of Florence, Florence, Italy

## Abstract

**Introduction:**

In this research, we investigate how Aberrant Salience (AS), Psychotic-Like Experiences (PLEs), and anxiety are interlinked in both healthy individuals and subjects with psychotic disorders. AS is a trait contributing to a susceptibility to psychosis and anxiety, while PLEs are subclinical states often leading to psychosis. We hypothesize that AS impacts the occurrence and severity of PLEs, which in turn influences anxiety.

**Objectives:**

The goal is to offer a more nuanced understanding of the risk factors leading to psychotic disorders and to shed light on anxiety psychopathogenesis in healthy and psychotic populations.

**Methods:**

We used self-reported questionnaires like the Aberrant Salience Inventory (ASI), Community Assessment of Psychic Experiences (CAPE), and Symptom Check List-90-revised (SCL-90-R). Data analysis included descriptive statistics and mediation analysis, adjusting for age, gender, and education. Controls were sourced through convenience and snowball sampling, while out-patients diagnosed with Schizophrenia Spectrum Disorder, Bipolar Disorder with psychotic features, or Major Depression with psychotic features were recruited from Florence University Hospital.

**Results:**

A total of 207 participants were included, with 163 controls and 44 patients. Descriptive statistics are shown in **Table 1.** Mediation analysis showed that PLEs frequency acted as a mediator between AS and anxiety only in the control group (**Figure 1**), not in patients (**Figure 2**).

**Table 1.** Descriptive statistics - Mean ± Std. Deviation.

	Control Group (N=163)	Psychotic Group (N=44)	p-value
ASI	11.690 ± 6.098	14.360 ± 7.163	**0.014**
CAPEposF	1.391 ± 0.340	1.617 ± 0.488	**0.001**
CAPEposD	1.792 ± 0.615	1.941 ± 0.694	0.167
SCL-90-R-ANX	0.678 ± 0.600	0.905 ± 0.643	**0.030**

**Legend:** ASI, Aberrant Salience Inventory; CAPEposF, Community Assessment of Psychic Experiences - positive dimension - Frequency; CAPEposD, Community Assessment of Psychic Experiences - positive dimension - Distress; SCL-90-R-ANX, Symptom Check List-90-revised, Anxiety.

**Image:**

**Figure:**
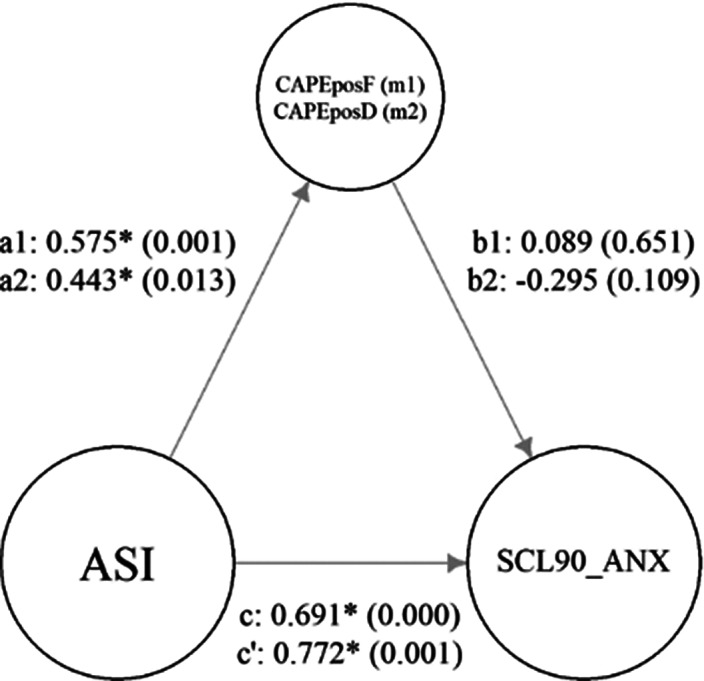

**Image 2:**

**Figure:**
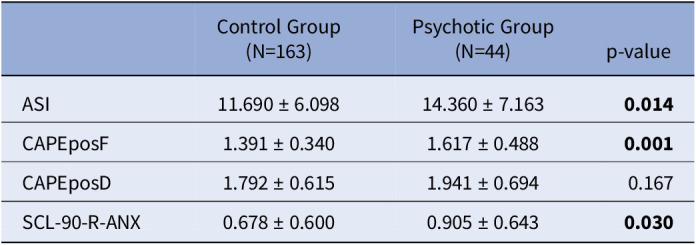

**Conclusions:**

PLEs triggered by AS led to anxiety in the control group but not in psychotic patients. The discrepancy could be due to reduced novelty and awareness of experiences in the patient group. This may affect how bodily responses to PLEs are perceived and suggests the need for specialized treatment approaches for anxiety in these two groups.

**Disclosure of Interest:**

None Declared

